# The validity of a portable clinical force plate in assessment of static postural control: concurrent validity study

**DOI:** 10.1186/2045-709X-20-15

**Published:** 2012-05-23

**Authors:** Samira Golriz, Jeffrey J Hebert, K Bo Foreman, Bruce F Walker

**Affiliations:** 1 School of Chiropractic and Sports Science, Murdoch University, 90 South Street, Murdoch, WA, 6150, Perth, Australia; 2 School of Chiropractic and Sports Science, Murdoch University, Perth, WA, Australia; 3 Department of Physical Therapy, University of Utah, Salt Lake City, UT, USA

**Keywords:** Concurrent validity, Force plate, Postural control

## Abstract

**Background:**

The broad use of force plates in clinical settings for postural control assessment suggests the need for instruments that are easy to use, affordable and readily available. In addition, these instruments of measurement should be reliable and valid as adequate reliability and validity are prerequisites to making correct inferences. The aim of this study was to examine the concurrent validity of postural control measures obtained with a clinical force plate.

**Methods:**

Thirty-one healthy adults were recruited. Participants completed 1 set of 5 trials on each force plate. Postural control measures (centre of pressure [COP] average velocity and sway area) were collected and compared using the Midot Posture Scale Analyzer (clinical force plate) and the Accugait force plate (criterion measure). Intra class correlation coefficient (ICC), standard error of measurement , and paired t-tests were calculated and Bland-Altman plots were constructed to compare the force plates and assess consistency of measurement and agreement between them.

**Results:**

The ICC values (ICC = 0.14-0.60) between the two force plates were lower than the acceptable value for both COP average velocity and sway area. There was significant difference (*p* > 0.05) in COP average velocity and sway area between the force plates. Examination of the plots revealed that there is less difference between the force plates in lower magnitudes of COP for average velocity and sway area however, the greater the average velocity and sway area, the greater the difference between the measures obtained from the two force plates.

**Conclusion:**

Findings of this study showed poor concurrent validity of the clinical force plate. This clinical force plate cannot be a replacement for known reliable and valid force plates and consequently measures obtained from this force plate should be treated with caution especially in a clinical population.

## Introduction

Postural control is a crucial factor in maintaining balance during standing, walking, task performance and when responding to the unexpected perturbations experienced in everyday life [1]. Postural control assessment can provide useful information when identifying individuals who are susceptible to postural control deficits [2,3]. Furthermore, postural control assessment has been used in sports medicine for selection of talented athletes, identification of athletes at high risk for injury, and for the prevention of sports related injuries [4]. Postural control is usually assessed by interpretation of parameters derived from the centre of pressure (COP) such as velocity and area of COP displacement [5]. COP is defined as the point of application of ground reaction forces under the feet.

In a clinical setting, force plates are regularly used to objectively assess postural control [6]. However, the reliability and validity of these force plates is often unknown. Reliability represents the consistency of measures or the level to which an instrument is free from errors of measurement [7]. Validity represents the extent to which an instrument measures what it is supposed to measure [7]. Concurrent validity examines the validity of an instrument against a criterion measure to test if the new instrument can be used instead of the criterion [7]. Understanding the reliability and validity of a measurement tool is crucial for making correct inferences from the data collected.

The Midot posture scale analyser (MPSA) is a lower cost portable force plate commonly used in the clinical setting. The MPSA has previously demonstrated acceptable reliability when averaging at least 5 values (ICC > 0.70) [8]. However, the validity of the MPSA has not been previously reported. Therefore, the purpose of this study was to examine the concurrent validity of postural control measures obtained with a clinical force plate when compared to a “gold standard” valid instrument.

## Materials and methods

### Participants

Participants were recruited through advertisements on University bulletin boards. The inclusion criteria were 18 to 60 years of age. A lower age limit of 18 years was set because the development of the skeletal system reaches its maturity at this age [9]. An upper age limit of 60 years was set because the ability to maintain postural control decreases in the elderly [10]. Potential participants were excluded from the study if they reported having balance deficits stemming from rheumatological or neurological disorders, a recent musculoskeletal injury (within three months), an ear infection or fever within 72 hours of the testing session, current pregnancy or the use of medications that could alter sensory perception. The Human Research Ethics Committee of Murdoch University approved the experimental protocol (2010/220), and all participants gave written consent before enrolment in the study.

### Instruments

The MPSA (QPS-200, Midot Medical Technology, Shekel Electronic Scale, Israel) is a portable force plate consisting of four electronic weighing plates set in a rectangular position. The MPSA records data using an internal sampling frequency of 200 Hz and filter frequency of 0.5 Hz. Calibration was conducted in accordance with the manufacturer’s recommendations using a 20 kg certified weight.

The Accugait (Advanced Mechanical Technology Inc., Watertown, MA,USA) was used as the criterion measure or reference standard. This force plate is a portable square force plate. There are flat rubber pads in each corner of the force plate to make the force plate less sensitive to vibration from most floor surfaces. The Accugait measures the three dimensional applied forces (Fx, Fy, Fz) and moments (Mx, My, Mz) involved in balance and uses established algorithms to compute the location of the COP and its associated variables from the forces and moments applied to the force plate. Data were acquired, recorded and analyzed using Balance Clinic software (balance software for AMTI’s Accusway plus balance platform, version 2.02.01) loaded on a Dell laptop.

**Figure 1:**
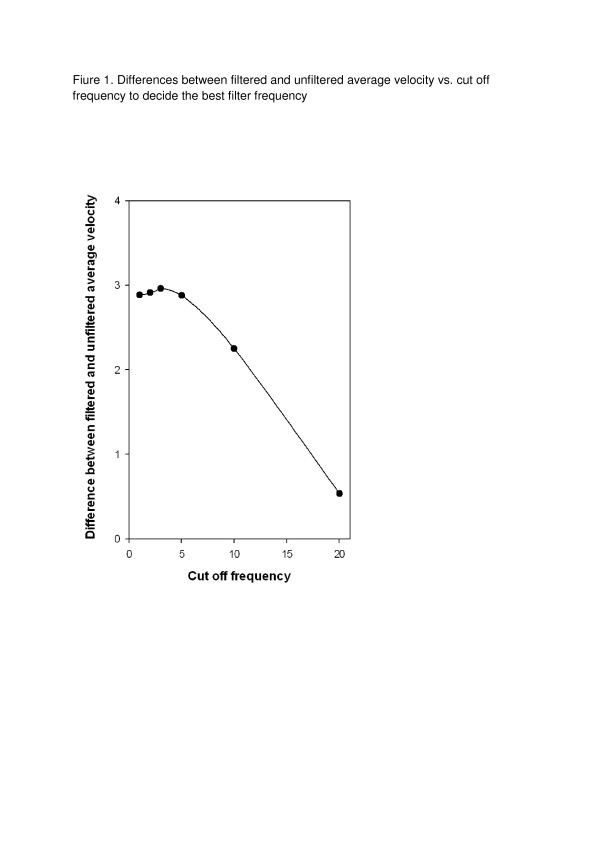
Differences between filtered and unfiltered average velocity vs. cut off frequency to decide the best filter frequency.

The Accugait force plate was initially factory validated prior to its release (personal communication with AMTI). However, to confirm the factory results we validated the Accugait based on the validation manual and validation report provided by AMTI [11]. The validation test represented an absolute COP error (the cumulative effect of noise and drift) of 3.6 mm for the X average, 3.4 mm for the Y average, 33.6 mm/s for average velocity and 2.9 mm^2^ for sway area over 40 seconds of data acquisition at a 50 Hz sampling frequency. All of these values were sufficiently close to the values reported in the AMTI validation report and as such the instrument was considered valid. We used data filtration to remove noise [12] and chose a 5Hz cut off frequency as the best level to filter data by performing a residual analysis. Residual analysis computes the differences between filtered and unfiltered signals over a wide range of cut off frequencies. To accomplish this we calculated differences between filtered and unfiltered COP average velocity signals over a wide range of cut off frequencies (1, 2, 3, 5, 10, 20 Hz) and plotted them (Figure 1). The plot was composed of linear and non linear parts. The linear part mostly represented random noise and the non linear part represented true signal. The cut off frequency where the plot turned from linear to non linear was chosen as the best cut off frequency for this experiment [12]. 

The acquisition sampling frequency of the Accugait was set at 100 Hz with the cut-off frequency of 5 Hz. Data was filtered using a fourth order Butterworth filter [13]. As we considered the Accugait as the criterion measure, the acquisition sampling frequency and cut off frequency of the Accugait was set at the optimal setting regarding the task and environment of our study. The force plate was calibrated with a calibration CD provided by the manufacturer and it was zeroed before each recording.

The COP average velocity (mm/sec) and the sway area, which is the area of an ellipse enclosing 95% of COP movements (mm^2^), are the two variables that were assessed and compared.

### Procedure

The two force plates were placed on the laboratory floor next to each other. The manufacturer recommends use of the force plate on any flat surface and adds there is no need to install the force plate permanently in the ground [13]. All participants were tested under the same conditions. Paper was placed on top of force plates and the force plates were zeroed. Participants were asked to remove their shoes and stand upright on the force plate and remain as still as possible with a relaxed posture. The participants were asked to put their arms to their sides in a comfortable position and distribute their body weight evenly on both feet. Also, they were asked to breathe normally and look straight ahead at an “X” on the opposite wall that was located two meters away at their eye level. Before commencement of data acquisition and after inspection for position symmetry, both feet were outlined on the piece of paper located on top of the force plate to ensure consistent foot placement across trials and between the two force plates. The same procedure was used for testing on the second force plate using the outline of the feet to ensure consistent foot placement.

Participants completed 1 set of 5 trials on each force plate as outlined above. To reduce the potential for bias due to ordering effects, we randomly allocated and counterbalanced the order in which the measurements were obtained on each force plate.

Five successive identical trials of 60 seconds were acquired on each force plate. Mandatory breaks of one minute and 5 minutes were allocated between trials and between force plates, respectively. Data were averaged across five trials, this number of repetitions was informed by a study where we assessed the reliability of the MPSA and found acceptable reliability when averaging at least 5 values [8].

### Statistical analysis

We conducted an *a priori* sample size estimation. Using the approach of Donner and Eliasziw (1987), assuming an alpha level of 0.05 and a minimally acceptable ICC value of 0.70 [7], recruitment of 30 participants provided 80% power [14].

Data management and statistical analyses were conducted using SPSS (version 17, Chicago, IL, USA). Data from 31 participants across 2 sets, 5 minutes apart were included for statistical analysis. Averages of 5 trials of COP average velocity and sway area were used for the analyses.

We calculated intraclass correlation coefficients (ICC_3,5_) for comparison between the two force plates. Paired t-tests were also performed to examine for differences in average velocity and sway area between the force plates. We examined the consistency of measurements between the 2 force plates by calculating the standard error of measurement using the formula: $\text{Standard error of measurement }=\text{ pooled standard deviation }\times \sqrt{1-\text{ICC}}$. Additionally, bias statistics with 95% confidence intervals and limits of agreement (LOA) were calculated and Bland-Altman plots [15] were constructed for COP average velocity and sway area. Bias represents the mean difference between the two force plates, while the LOA examines agreement between the two force plates. LOAs are defined as bias ± 1.96 SD, where SD is the standard deviation of the difference between measures of the two force plates.

## Results

Thirty-one healthy participants consisting of 18 males and 13 females, aged 23 to 58 were recruited from the staff and students of Murdoch University. All participants were able to complete all test repetitions and all data were included for statistical analysis. The mean (SD) age of the participants was 32.7 (8.5) years and BMI was 24.7 (5.4). Descriptive statistics, ICC, standard error of measurement, bias and limits of agreement are presented in Table 1 for average velocity and sway area. The ICC values between the two force plates are lower than the acceptable value with wide confidence intervals for both average velocity and sway area. Paired t-tests showed significant differences in average velocity and sway area between the 2 force plates (*p* < 0.05).

**Table 1:** Criterion validity analysis

	**Mean(SD)**	**t-test** **(p value)**	**SEM**	**ICC**_**(3,5)**_**(CI’s)**	**Bias (95% CI) ± 95% LOA**
**COP average velocity (mm/s)**					
Accugait	8.3 (2.3)				
		0.004*	2	0.60(0.16- 0.80)	1.7† (1.1,2.2) ± 5.9
MPSA	6.6 (3.5)				
**Sway area (mm**^**2**^**)**					
Accugait	274 (155)				
		0.000*	870	0.14 (−0.24- 0.48)	−1660† (−1370,- 1950) ± 3134
MPSA	1936 (1720)				

CI, confidence Interval; *p < 0.05 indicates a significant difference in values between the 2 force plates; †statistically significant bias (different from zero).

**Figure 2:**
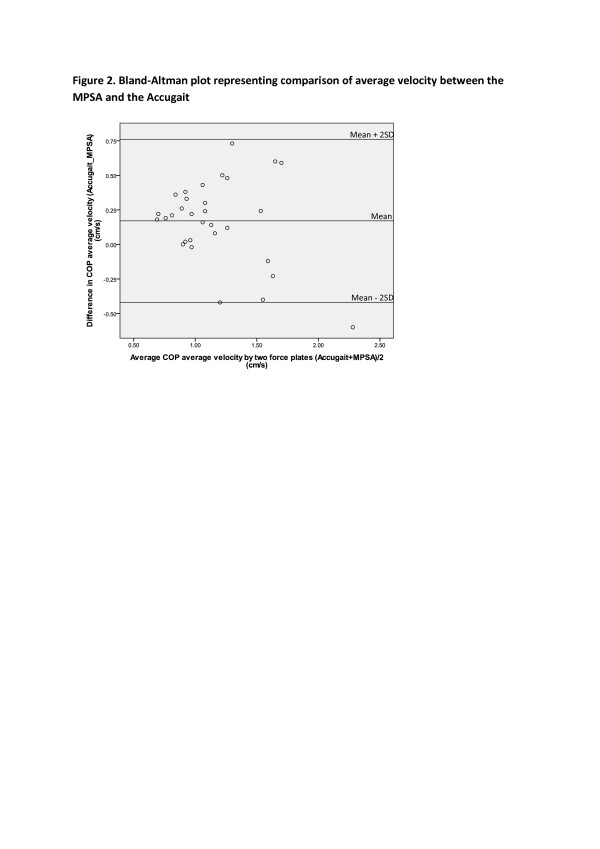
Bland-Altman plot representing comparison of average velocity between the MPSA and the Accugait.

**Figure 3:**
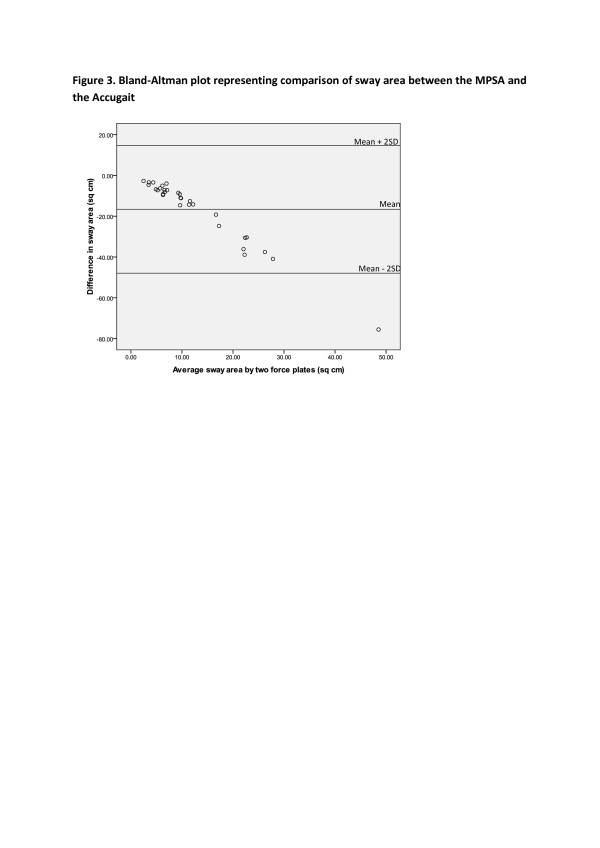
Bland-Altman plot representing comparison of sway area between the MPSA and the Accugait.

The Bland–Altman plots for the average velocity and sway area are provided in Figures 2 and 3 and demonstrate more variation between the measures obtained from the two force plates when the amount of COP average velocity and sway area were higher.

## Discussion

The purpose of this study was to investigate the concurrent validity of the MPSA by measuring COP variables and comparing these with a validated reference standard. The ICCs of the postural control measures were equal to 0.60 for the average velocity and 0.14 for the sway area between the force plates. Results of the reliability study of the MPSA also reported higher agreement for the average velocity and a lower agreement for sway area [8]. The ICC is a ratio estimate and there is no widely agreed upon thresholds for identifying an acceptable level of agreement with ICC reporting. As a result, there are various interpretations of ICC values available in the literature. Landis and Koch [16] suggested the following qualitative approach to ICC interpretation: 0.00-0.20 slight, 0.21-0.40 fair, 0.41-0.60 moderate, 0.61-0.80 substantial and 0.81-1.00 almost perfect. Alternatively, Portney and Watkins [7] suggest that values over 0.75 indicate good agreement, and values below 0.75 are indicative of poor to moderate agreement. The highest level of agreement identified in the current study was ICC = 0.60. Therefore, these results represent evidence that the MPSA does not possess sufficient concurrent validity.

The high standard error of measurement values of both variables especially the sway area, when compared to the mean values obtained by the two force plates, indicates a lack of consistency of measurement between the force plates. Furthermore, our results demonstrated significant differences between the COP measurements obtained from the force plates for the COP average velocity and sway area.

Comparison of standard deviations to means of sway area captured by the two force plates shows that the large standard deviation of MPSA sway area indicates a high degree of random error in the MPSA data. Additionally, the large mean difference between MPSA and Accugait sway area might be a sign of a systematic error in the MPSA when assessing postural control.

Visual inspection of the Bland-Altman plot (Figure 2) of average velocity shows a funnel effect, meaning there is more variability when the magnitude of the average velocity is greater. Our results represent excessive variation between the force plates in higher magnitudes of average velocity and less variation in lower magnitudes of average velocity. Differences between the measures of COP velocity between the force plates depend on the magnitude of the measurement. The excessive variations in higher magnitudes of average velocity indicate that data captured by the MPSA contain errors for measuring higher COP velocities.

The bias estimates for COP velocity were small but statistically different from zero. Statistically significant bias was found for the average velocity variable where the MPSA values underestimate the criterion in most of the cases with an estimate of 1.7 and a 95% confidence interval of 1.1 - 2.2. One possible explanation for this is that data captured by the Accugait was filtered with a 5 Hz cut off frequency whereas data obtained by the MPSA was filtered with a fixed 0.5 Hz cut off frequency. This may have resulted in the MPSA eliminating true signal. The lower cut off frequency causes removing higher proportion of true signals.

The plot of the sway area (Figure 3) shows a systematic trend. With lower magnitudes of sway area the differences between the two force plates are relatively low as compared to higher magnitudes, but the greater the sway area the bigger the difference between the two force plate measures. Moreover, the statistically significant bias of sway area showed that the MPSA overestimated sway area. Overall, larger measures of average velocity and sway area result in increased systematic and random error in MPSA.

While the MPSA previously demonstrated acceptable reliability [8], its validity is not satisfactory; therefore, it cannot be considered a replacement of a known valid force plate for the assessment of postural control. In addition, results obtained by the MPSA on clinical populations should be treated with caution. Clinical populations are potentially less stable and show higher magnitudes of average velocity and sway area [17,18] and the MPSA was incapable of measuring accurate data with higher magnitudes of average velocities and sway areas. Additionally, to reduce measurement error, we used a mean of 5 trials, which is unlikely to be the case with clinical use given the time constraints of clinical practice. Clinicians should consider all the above mentioned issues when assessing postural control using the MPSA.

Although we cannot rely on the MPSA for assessment of postural control, it may possibly be used in obtaining qualitative estimates of postural control, for instance as a biofeedback training tool and to enhance motivation level of patients with balance defects. Having said that, we suggest clinicians intending to use a force plate purchase a reliable and valid instrument.

Limitations exist within this study. The study sample consisted of healthy individuals and the findings may or may not generalize to clinical populations. This study did not attempt to assess the validity of the MPSA in different testing positions such as eyes closed, single limb standing and narrow stance (feet together) although we hypothesis that this would not enhance the MPSA performance.

Future research could be done to assess the validity of the MPSA in different balance testing positions and clinical populations. Estimates of reliability and validity should be known prior to using any type of force plates.

In conclusion, postural control parameters cannot be validly measured in clinical or research settings using the MPSA. The MPSA is a lower cost force plate with a low-technology design and easy to use software but it did not fulfil the criteria to be regarded as a valid force plate for clinical use.

## Abbreviations

COP,Centre of pressure; ICC,Intraclass correlation coefficient; LOA,Level of agreement; MPSA,Midot posture scale analyser.

## Competing interests

The authors declare that they have no competing interests.

## Authors’ contributions

SG participated in the design of the study, collected data, performed the statistical analysis and drafted the manuscript. JH participated in the design of the study, helped to perform the statistical analysis and drafted the manuscript. BF helped to draft the manuscript. BW participated in the design of the study and helped to draft the manuscript. All authors read and approved the final manuscript.
